# Poor Embryo Quality Is Associated With A Higher Risk of Low Birthweight in Vitrified-Warmed Single Embryo Transfer Cycles

**DOI:** 10.3389/fphys.2020.00415

**Published:** 2020-05-15

**Authors:** Jiaan Huang, Yu Tao, Jie Zhang, Xiaoyan Yang, Jiayi Wu, Yanping Kuang, Yun Wang

**Affiliations:** Department of Assisted Reproduction, Shanghai Ninth People’s Hospital, Shanghai Jiao Tong University School of Medicine, Shanghai, China

**Keywords:** embryo quality, single embryo transfer, neonatal outcomes, maternal outcomes, vitrified–warmed embryo transfer

## Abstract

**Background:**

Previous studies have reported the association between embryo quality and perinatal outcomes in fresh cycles, after cleavage-stage or blastocyst embryo transfer, and found no significant difference. However, in terms of vitrified-warmed embryo transfer cycles, the impact of embryo quality on neonatal and maternal outcomes has not been evaluated.

**Objectives:**

To explore the association between the quality of a single vitrified-warmed embryo and perinatal outcomes.

**Methods:**

This retrospective study included 2403 live-born singletons derived from single vitrified-warmed embryo transfer cycles during January 2006 and July 2018. Neonatal and maternal outcomes were compared between singletons resulting from the use of single good quality embryo (GQE) (*n* = 1854) and single poor quality embryo (PQE) (*n* = 549) and analyzed in the group of cleavage-stage embryo transfer and the group of blastocyst transfer, respectively.

**Results:**

A significantly higher risk of low birthweight (LBW, birthweight <2500 g) was observed in the singletons derived from the transfer of single PQE compared with those derived from the transfer of single GQE both in cleavage and blastocyst stages (cleavage-stage, AOR 2.62, 95% CI 1.27–5.37; blastocyst stage, AOR 1.98, 95% CI 1.06–3.70). An increased risk of preterm birth (PTB, gestational age <37 weeks) was also observed in singletons born after transfer of a PQE of cleavage-stage compared with those after a GQE of cleavage-stage (AOR 2.40, 95% CI 1.28–4.49). The transfer of single poor quality blastocyst was associated with a higher risk of placenta previa compared with the transfer of single good quality blastocyst (AOR 2.65, 95% CI 1.26–5.57). Other maternal complications, neonatal malformations, and neonatal complications were similar between compared groups.

**Conclusion:**

In vitrified-warmed cycles with single embryo transfer, poor embryo quality would result in a significantly higher risk of LBW, regardless of cleavage-stage or blastocyst embryo transfer. Meanwhile, the transfer of poor cleavage-stage embryo was also associated with an increased incidence of PTB.

## Introduction

The technology of cryopreservation has been refined by the use of vitrification and therefore optimized the process of frozen embryo transfer (FET). In recent years, FET has become a viable alternative to fresh embryo transfer in *in vitro* fertilization (IVF) attempts, along with an increasingly suggested freeze-all strategy, which has shown increased pregnancy rates and minimized risk of ovarian hyperstimulation syndrome (OHSS) (Zhu et al., 2018; Stormlund et al., 2019). Since the introduction of the embryo morphological assessment system, embryo quality has served as an important guide for successful implantation and live birth in embryo transfer cycles, especially for blastocyst (Ahlstrom et al., 2011; Bouillon et al., 2017). Recently, a 1.0–3.5-fold increased incidence of clinical implantation has been reported after the transfer of GQE compared to PQE in fresh cycles (Oron et al., 2014a, b; Zhu et al., 2014; Bouillon et al., 2017; Dobson et al., 2018; Irani et al., 2019). Interestingly, previous studies involving fresh cycles found no difference between singletons born after the transfer of PQE and those born after GQE in risks of LBW, PTB, small for gestational age (SGA) and other adverse perinatal outcomes (Oron et al., 2014a, b; Zhu et al., 2014; Bouillon et al., 2017). Despite these reassuring findings, patients with only poor embryos available for transfer were likely to be predisposed to worse prognosis with the underlying pathology of decreased oocyte quality or poor response to ovarian stimulation (van Rooij et al., 2003; Check et al., 2007; Morin et al., 2018). Of mounting concerns, however, it remains unknown whether the quality of the single vitrified-warmed embryo is associated with neonatal and maternal outcomes.

It has been reported that the risks of LBW, PTB, and birth defects were more common in singletons conceived by assisted reproductive technology (ART), compared with sibling pairs without ART (Pinborg et al., 2013; Martin et al., 2017). However, in terms of FET, a better, or equal perinatal outcome has been well established compared with fresh embryo transfer (Pelkonen et al., 2010; Maheshwari et al., 2018; Sha et al., 2018), of those studies, some of them hypothesized that better fetal growth resulting from FET may be due to the selective bias of GQEs in the cryopreservation process (Pelkonen et al., 2010; Maheshwari et al., 2018). Additionally, more and more studies have found higher birthweight and a higher proportion of large for gestational age (LGA) after blastocyst transfer compared with cleavage-stage embryo transfer (Makinen et al., 2013; Zhang et al., 2019).

As a result, the prime end-point of embryo quality assessment is the live birth of a healthy baby, therefore, the aim of our study was to evaluate the impact of embryo quality on the neonatal and maternal outcomes of singleton live births born after single cleavage-stage or blastocyst embryo transfer following FET, after adjusting for potential confounding variables.

## Materials and Methods

### Study Population and Design

We retrospectively evaluated 2403 infertile women who delivered live-born singletons originating from single vitrified-warmed embryo transfer, after IVF or intracytoplasmic sperm injection (ICSI), between January 2006 to July 2018 at the Department of Assisted Reproduction of Shanghai Ninth People’s Hospital, Shanghai Jiao Tong University School of Medicine. The inclusion criteria were: (i) cycles after single vitrified-warmed embryo transfer, (ii) patients ≤40 years of age with a BMI ≤30 kg/m^2^, and (iii) the first singleton born alive after the 20th week of gestation with ART. Furthermore, patients with vanishing twin were excluded, as was any use of PGD and assisted hatching. Women were included only once in the study.

### Analysis

A total of 8407 infertile women were included during the study period, of which 2403 live-born singletons were delivered. First, the association between embryo quality and pregnancy outcomes was assessed in 8407 embryo transfer cycles included in our study. In the second part, we performed the analysis between live births resulted from single PQE of cleavage-stage (*n* = 175) versus single GQE of cleavage-stage (*n* = 922) and single PQE of blastocyst (*n* = 374) versus single GQE of blastocyst (*n* = 932), respectively. The GQE group was the reference group. Logistic regression analysis was used to adjust for patient and FET characteristics including maternal age (categorized as [years] <35, 35–37, and 38–40), maternal BMI (continuous variable), parity (primiparity yes/no), FET rank (first FET rank or higher order FET rank), number of total oocytes retrieved (continuous variable), number of Metaphase II (MII) oocytes (continuous variable), etiology of infertility [tubal factor infertility (including salpingitis history, unilateral or bilateral tubal obstruction, and the presence of tubal adhesion or hydrosalpinx), polycystic ovary syndrome (PCOS) (Rotterdam Eshre/Asrm-Sponsored Pcos Consensus Workshop Group., 2004), poor ovarian reserve (defined by either a basal antral follicle count ≤4 or a serum FSH level ≥11.4 mIU/ml on day 3) (Molinaro et al., 2009), male infertility (oligoasthenoteratozoospermia or an azoospermia:sperm concentration <15 × 10^6^/mL, vitality <40%, motility <32%, normal morphology <4%) (World Health Organization, 2010) and other causes including uterine factor, endometriosis and unexplained infertility], method of endometrial preparation (natural cycles, stimulated cycles and hormone therapy cycles), endometrial thickness on embryo transfer (ET) day [categorized as (mm) <8, 8–14, and >14], day of embryo transfer (day 3, day 5, and day 6), year of treatment (2006–2012, 2013–2014, and 2014–2017) and newborn gender (male or female).

The follow-up system in our study has been previously described (Chen et al., 2015; Du et al., 2018). Briefly, the couples completed the interview questionnaires mainly by the telephone inquiry, and the interviews were conducted from the time confirming clinical pregnancy, till 1 week after delivery. Abnormal situations including serious pregnancy complications, congenital malformations, and neonatal complications were further questioned, and the necessary information was obtained from the responsible doctors.

### Laboratory Protocols and Embryo Quality

Details on protocols of controlled ovarian hyperstimulation, method of endometrial preparation and procedures of IVF/ICSI have been described previously (Chen et al., 2015; Du et al., 2018; Zhang et al., 2019). In brief, regarding semen analysis results and previous infertility characteristics, IVF or ICSI was performed 3–6 h after oocyte retrieval. For IVF, collected oocytes were incubated in human tubal fluid (HTF; Irvine Scientific, United States) supplemented with 10% serum substitute supplement (SSS; Irvine Scientific, United States) and 300,000 progressively motile spermatozoa and left overnight. For ICSI, denudated oocytes were injected with a single mechanically immobilized sperm and directly thereafter cultured in fertilization medium (HTF+10% SSS). Notably, from 2013 onward, all embryos were maintained in Continuous Single Culture of HTF (Irvine Scientific, United States) throughout the entire duration of *in vitro* culture, however, before 2013, embryos were cultured in early cleavage medium (Irvine Scientific, United States) before day 3, and in Multiblast Medium (Irvine Scientific, United States) sequentially. All embryos were incubated under oil at 37°C and a 5% O2 and 6% CO2 environment. Eventually, successful fertilization was indicated by the distinct appearance of two pronuclei 16–18 h after IVF/ICSI. And then, the embryonic development and morphology were assessed on day 3 for the first time.

Evaluation of cleavage-stage embryos was based on the number of blastomeres, blastomere size, fragmentation rate, and the presence of multinucleated blastomeres (Reinblatt et al., 2011). We defined cleavage-stage embryos as good quality if there were 7,8 cells on day 3, contained <20% anucleate fragments, and presented no apparent multinucleated blastomeres. PQE of cleavage-stage covered two grades, including fair quality embryos, with only 3–5 cells on day 3 and/or 20–50% fragmentation and poor quality embryos as those with <3 cells and/or >50% fragmentation by day 3. On day 5/6, embryos were assessed according to the expansion of the blastocoel cavity (B1–B6), and the number and cohesiveness of the inner cell mass (ICM) and trophectodermal cells (TE) (Gardner and Schoolcraft, 1999). In detail, the degree of expansion was graded as [B3–B6] if the blastocele fills the entire blastocyst onward; The ICM was graded as [B] if the blastocyst loosely gathered cells; The TE was graded as [B] if few uneven cells creating a loose epithelium in the embryo. [A] performed superiorly than [B] with more cells in both ICM and TE and [C] ranked last. In short, GQE of blastocyst including blastocysts showed B3–B6 with at least [B] for the ICM and the TE. PQE of blastocyst showed B3–B6 with at least [C] for ICM or [C] for the trophectoderm. All the embryos grading was carried out by one of the three trained embryologists, each with over 10 years of experience. The extended culture of embryos was determined according to the quantity and quality of cleavage-stage embryos, combined with the request of patients and the evaluation of clinicians.

We used the quality of embryos post-thawed as the variable in our study, which was the same as the embryo quality before vitrification in our study. The vitrification process was performed using the Cryotop carrier system combined with dimethylsulfoxide–ethylene glycol–sucrose as the cryoprotectants. Based on the number and quality, a day 3, day 5, or day 6 embryo was thawed via dilution solution in a sequential manner (1 mol/L to 0.5 mol/L to 0 mol/L sucrose), after which, the embryo quality was graded once again and then transferred back into the uterus. For all embryos, the survival rate was over 99% in our study, and the time from warming to transfer was 3–4 h.

### Outcome Measures

Neonatal outcomes were the following: gestational age (calculated from the day of embryo transfer according to embryo stage and adjusted by ultrasonographic assessment), birthweight, z-scores (birthweight after adjusted for gestational age and newborn gender), low birth weight (LBW, birthweights <2500 g), very low birth weight (VLBW, birthweights <1500 g), and high birth weight (HBW, birthweights >4500 g), preterm birth (PTB, gestational age <37 weeks) and very preterm birth (VPTB, gestational age <32 weeks), small for gestational age (SGA, birthweights <10th percentiles) and very small for gestational age (VSGA, birthweights <3rd percentiles), LGA (birthweights >90th percentiles) and very large for gestational age (VLGA, birthweights >97th percentiles). Congenital malformations were classified under the guidance of the International Classification of Diseases (ICD) Q codes (Q00–Q99) as conditions registered in the International Statistical Classification of Diseases and Related Health Problems. Neonatal complications included respiratory or gastrointestinal complications and admission to the neonatal intensive care unit (NICU). Birthweight percentiles and calculation of Z-scores were based on Chinese reference singleton newborns stratified by gestational age and neonatal sex (Dai et al., 2014). Maternal complications included gestational diabetes (GDM) (ICD 10 code O24.4), pregnancy-induced hypertension (PIH) (ICD 10 code O13-14), preterm premature rupture of membranes (PPROM) (ICD 10 code O42.2–49.9), pre-eclampsia (ICD 10 code O13–15), placenta previa (ICD 10 code O44.0–44.1), and post-partum hemorrhage (ICD 10 code O72).

### Statistical Analyses

Statistical analysis was performed by SPSS version 24.0 software (SPSS Inc., Tsinghua, China). Continuous variables for patient baseline characteristics were assessed for normality graphically using the Shapiro–Wilk test combined with the frequency distribution curve and expressed as mean (±SD) or median (interquartile range) according to if variables were normally distributed or not. Categorical variables were presented with their frequency and percentage within the study group. Between-group differences were assessed using the *t*-test or Mann–Whitney–Wilcoxon tests for continuous variables, and comparisons of rates were performed by the chi-square test or Fisher’s exact test as appropriate, and a Pearson correlation coefficient was provided for correlations. Potential confounding factors including maternal age, maternal BMI, parity, FET rank, number of total oocytes retrieved, number of MII oocytes, etiology of infertility, method of endometrial preparation, endometrial thickness on ET day, day of embryo transfer, year of treatment and newborn gender. Only one multiple logistic regression model was performed in the analysis. A two-sided *P*-value <0.05 was considered to determine statistical significance.

## Results

A total of 2403 live births born after vitrified-warmed SET cycles were enrolled. There were 1097 patients with cleavage-stage embryo transferred who delivered singletons, including 922 GQE transfer cycles and 175 PQE transfer cycles. In blastocyst embryo transfer cycles, there were 1306 of them delivered singletons including 932 GQE transfer cycles and 374 PQE transfer cycles. Every patient gave birth to one live-born singleton in this study. The pregnancy outcome of included cycles are summarized in Table 1. A significant difference existed in clinical pregnancy and live birth rates between PQE and GQE (*P* < 0.01), both in the embryo stage of cleavage (23.7% and 19.5% vs 30.5% and 24.5%, respectively) and blastocyst stage (35.7% and 29.5% vs 48.6% and 40.2%, respectively). Of the singleton clinical pregnancies achieved after vitrified-warmed SET cycles, the rates of miscarriages, ectopic pregnancies, stillbirths, and live births were comparable between GQE and PQE groups.

**TABLE 1:** Pregnancy outcome of vitrified-warmed SET cycles stratified into four groups according to embryo quality and embryo stage.

	**GQE of cleavage (*n* = 3815)**	**PQE of cleavage (*n* = 916)**	**GQE of blastocyst (*n* = 2364)**	**PQE of blastocyst (*n* = 1312)**	***P-value^a^***	***P-value^b^***
Clinical pregnancies^c^	1165(30.5)	217(23.7)	1148(48.6)	485(35.7)	<*0.001*	<*0.001*
Live births^c^	934(24.5)	179(19.5)	950(40.2)	387(29.5)	0.002	<*0.001*
Pregnancy outcome out of singleton clinical pregnancies	*n* = 1146	*n* = 212	*n* = 1116	*n* = 454		
Miscarriages	202(17.6)	35(16.5)	170(15.2)	75(16.5)	0.694	0.524
Ectopic	18(1.6)	1(0.5)	11(1.0)	4(0.9)	0.340	0.844
Stillbirths	4(0.3)	1(0.5)	3(0.3)	1(0.2)	0.573	0.863
Live births	922(80.5)	175(82.5)	932(83.5)	374(82.4)	0.477	0.586

*Clinical pregnancy was diagnosed by the ultrasonographic visualization of one or more gestational sac after embryo transfer; Miscarriage rate was defined as a loss of clinical pregnancy before the 20th gestational week; Data are were presented as numbers and and proportion (%). ^*a*^*P*-value for PQE of cleavage vs GQE of cleavage. ^*b*^*P*-value for PQE of blastocyst vs GQE of blastocyst. ^*c*^Inclusion of single and twin pregnancies.*

Patient and cycle characteristics are illustrated in Table 2. Maternal age, BMI, parity, and endometrial thickness were similarly distributed in groups. The numbers of total oocytes collected and MII oocytes were significantly higher in the GQE group of blastocyst stage. There were significantly more later developing blastocyst of day 6 embryo amongst the live births from the transfer of single PQE of the blastocyst (61.4% vs 79.9%, respectively; *P* < 0.001).

**TABLE 2:** Patient and cycle characteristics.

**Characteristics**	**GQE of cleavage (*n* = 922)**	**PQE of cleavage (*n* = 175)**	**GQE of blastocyst (*n* = 932)**	**PQE of blastocyst (*n* = 374)**	***P-value^a^***	***P-value^b^***
Maternal age (years)*	31.5 ± 3.9	31.6 ± 4.0	31.0 ± 3.9	31.7 ± 3.8	0.596	0.116
<35	717(77.8)	130(74.2)	752(80.7)	286(76.5)		
35–37	139(15.1)	31(17.7)	125(13.4)	55(14.7)		
38–40	66(7.2)	14(8.0)	55(5.9)	33(8.8)		
Maternal BMI (kg/m^2^)	21.7 ± 2.9	21.9 ± 2.6	21.4 ± 2.7	21.3 ± 2.5	0.404	0.357
Duration of infertility(years)	3.6 ± 2.9	3.5 ± 3.0	3.3 ± 2.7	3.4 ± 2.9	0.571	0.452
Primiparity(%)^c^	847(91.9)	156(89.1)	871(93.5)	342(91.4)	0.238	0.201
FET rank(%)^c^					0.674	0.584
1	474(51.4)	93(53.1)	349(37.4)	134(35.8)		
≥2	448(48.6)	82(46.9)	583(62.6)	240(64.2)		
Main infertility cause (%)^c^					0.685	0.845
Tubal factor infertility	483(52.4)	89(50.9)	554(59.4)	224(59.9)		
PCOS	75(8.1)	15(8.6)	81(8.7)	21(5.6)		
Poor ovarian reserve	49(5.3)	14(8.0)	23(2.5)	14(3.7)		
Male infertility	171(18.5)	29(16.6)	131(14.1)	67(17.9)		
Other	144(15.6)	28(16.0)	143(15.3)	48(12.8)		
Endometrial preparation (%)					0.150	0.273
Natural cycles	240(26.0)	40(22.9)	252(27.0)	85(22.7)		
Hormone therapy cycles	329(35.7)	76(43.4)	350(37.6)	148(39.6)		
Stimulated cycles	353(38.3)	59(33.7)	330(35.4)	141(37.7)		
Endometrial thickness on FET day*	10.8 ± 2.2	10.3 ± 2.0	10.7 ± 2.5	10.5 ± 2.4	0.488	0.843
<8.0	65(7.0)	12(6.9)	90(9.7)	40(10.7)		
8.0–14.0	780(84.6)	153(87.4)	759(81.4)	302(80.7)		
>14.0	77(8.4)	10(5.7)	83(8.9)	32(8.6)		
Oocytes per cycle						
Total^c^	7.1 ± 5.9	7.0 ± 6.1	10.6 ± 7.2	8.9 ± 6.2	0.257	<*0.001*
Metaphase II^c^	5.7 ± 4.9	5.4 ± 5.0	8.5 ± 5.7	7.3 ± 5.1	0.116	<*0.001*
Fertilized (%)	67.1	64.8	68.6	67.6	0.765	0.897
Procedure					0.339	0.402
IVF	593(64.3)	108(61.7)	616(66.1)	233(62.3)		
ICSI	235(25.5)	53(30.3)	228(24.5)	104(27.8)		
IVF+ICSI	94(10.2)	14(8.0)	88(9.4)	37(9.9)		
Day of embryo transfer ^c^					-	<*0.001*
Day 3	922(100)	175(100)				
Day 5			375(38.6)	79(20.3)		
Day 6			596(61.4)	310(79.7)		
Year of treatment (%)^c^					<*0.001*	<*0.001*
2006–2012	119(12.9)	10(5.7)	152(16.3)	55(14.7)		
2013–2014	360(39.0)	7(4.0)	291(31.2)	73(19.5)		
2015–2018	443(48.0)	159(90.3)	489(52.4)	246(65.7)		

*Data are presented as mean (±SD) or median (interquartile range) according to if variables were normally distributed or not. Continuous variables were compared using *t*-test or Mann–Whitney–Wilcoxon tests, and categorical variables were presented as numbers (%) and evaluated with chi-squared tests. ^*a*^*P*-value for PQE of cleavage vs GQE of cleavage. ^*b*^*P*-value for PQE of blastocyst vs GQE of blastocyst. ^*c*^*P* < 0.05 for PQE vs GQE in all patients. ^∗^*P*-value of the data were analyzed as categorical variable.*

Descriptive statistics for the neonatal and maternal outcomes are illustrated in Table 3. An increased risk of LBW was found in the group of PQE compared to the group of GQE in both embryo stage of cleavage (8.0% vs 3.1%, *p* = 0.002) and blastocyst (5.9% vs 2.7%, *p* = 0.005), while the rates of VLBW (for cleavage-stage, 1.1% vs 0.5%; for blastocyst, 0.8% vs 0.6%) were comparable between the GQE and PQE groups. The proportion of PTB was significantly higher in the PQE of cleavage group (10.9% vs 6.3%, *p* = 0.030), while no significant difference was found in the PQE of blastocyst stage (8.0% vs 7.4%). Although the rate of VPTB in the PQE group was double that in the GQE group (for cleavage-stage, 0.5% vs 1.7%; for blastocyst, 0.6% vs 1.1%), the difference was not statistically significant. The transfer of single poor quality blastocyst was associated with a higher risk of placenta previa compared with the transfer of single good quality blastocyst (4.5% vs 2.3%, *p* = 0.026). The other maternal complications were not significantly correlated with embryo quality.

**TABLE 3:** Neonatal and maternal characteristics of live born singletons.

**Characteristics**	**GQE of cleavage (*n* = 922)**	**PQE of cleavage (*n* = 175)**	**GQE of blastocyst (*n* = 932)**	**PQE of blastocyst (*n* = 374)**	***P-value^a^***	***P-value^b^***
Gestational age (weeks)						
<32 weeks	5(0.5)	3(1.7)	6(0.6)	4(1.1)	0.121	0.485
32–36 weeks	53(5.7)	16(9.2)	63(6.8)	26(7.0)	0.090	0.901
≥37 weeks	864(93.7)	156(89.1)	863(92.6)	344(92.0)	0.030	0.703
Mode of delivery (%)					0.040	0.407
Vaginal	203(22.0)	51(29.1)	219(23.5)	96(25.7)		
Caesarean section	719(78.0)	124(70.9)	713(76.5)	278(74.3)		
Sex (%)					0.794	<0.001
Male	459(49.8)	89(50.9)	556(59.7)	187(50.0)		
Female	463(50.2)	86(49.1)	376(40.3)	187(50.0)		
Birthweight (g)^*d*^	3295.5 ± 478.5	3279.8±545.1	3386.5 ± 492.5	3330.4±498.9	0.722	0.004
Z-score	0.26 ± 1.03	0.27 ± 0.99	0.51 ± 1.04	0.35 ± 1.10	0.889	0.011
2500–4500 g^c^	887(96.2)	161(92.0)	898(96.4)	346(92.5)	0.014	0.003
VLBW(<1500 g)	5(0.5)	2(1.1)	6(0.6)	3(0.8)	0.310	0.721
LBW(<2500g)^c^	29(3.1)	14(8.0)	25(2.7)	22(5.9)	0.002	0.005
HBW(>4500 g)	6(0.7)	1(0.6)	9(1.0)	6(1.6)	0.904	0.489
SGA(<10th percentile)	60(6.5)	7(4.0)	33(3.5)	22(5.9)	0.204	0.057
VSGA(<3th percentile)	16(1.7)	3(1.7)	7(0.8)	7(1.9)	0.984	0.139
LGA(>90th percentile)	135(14.6)	21(12.0)	207(22.2)	71(19.0)	0.359	0.198
VLGA(>97th percentile)	43(4.7)	10(5.7)	82(8.8)	26(7.0)	0.552	0.273
Congenital	9(1.0)	3(1.7)	9(1.0)	7(1.9)	0.420	0.176
malformations (%)						
Neonatal	45(4.9)	9(5.1)	39(4.2)	23(6.1)	0.883	0.131
Complications (%)						
Maternal						
Complications (%)						
GDM	102(11.0)	17(9.7)	98(10.5)	51(13.6)	0.599	0.109
PPROM	12(1.3)	5(2.9)	15(1.6)	9(2.4)	0.170	0.332
Placenta previa	28(3.0)	5(2.9)	21(2.3)	17(4.5)	0.898	0.026
PIH	31(3.4)	4(2.3)	27(2.8)	9(2.4)	0.485	0.624
Pre-eclampsia	17(1.8)	3(1.7)	14(1.5)	7(1.9)	0.907	0.631
Post-partum haemorrahage	15(1.6)	4(2.3)	17(1.8)	5(1.3)	0.527	0.536

*GDM = gestational diabetes; PPROM = preterm premature rupture of membranes; PIH = pregnancy-induced hypertension; Data are presented as mean (±SD) or median (interquartile range) according to if variables were normally distributed or not. Continuous variables were compared using *t*-test or Mann–Whitney–Wilcoxon tests, and categorical variables were presented as numbers (%) and evaluated with chi-squared tests. ^*a*^*P*-value for PQE of cleavage vs GQE of cleavage. ^*b*^*P*-value for GQE of blastocyst vs GQE of blastocyst. ^*c*^*P*-value < 0.001 for PQE vs GQE in all singletons. ^*d*^*P*-value = 0.045 for PQE vs GQE in all singletons.*

Table 4 presents the results of adverse neonatal and maternal outcomes after adjustment for risk factors. For singletons born in PQE groups, the increased risk of LBW remained significant compared to those in GQE groups. In the group of cleavage stage, not only an increased risk of LBW but also a higher risk of PTB was observed in the group of PQE in comparison to the group of GQE. In the group of blastocyst, women with PQE transferred were at a higher risk of placenta previa. The types of congenital malformations among live-born singletons in our study were shown in Supplementary Table S1. We evaluated the associations between embryo quality and very PTB, VLBW HBW, SGA, VSGA, LGA, VLGA, neonatal complications, and congenital malformations, but no significant differences were detected (*P* > 0.05).

**TABLE 4:** COR and AORs in adverse neonatal and maternal outcomes of live born singletons born after GQE and PQE transfer stratified by embryo stage of cleavage and blastocyst.

	**PQE of cleavage vs GQE of cleavage**			**PQE of blastocyst vs GQE of blastocyst**		
**Outcome**	**COR (95%CI)**	**AOR (95%CI)**	***P***	**COR (95%CI)**	**AOR (95%CI)**	***P***
VPTB(<32 weeks)	3.20(0.76,13.51)	5.04(0.94,26.99)	0.059	1.67(0.47,5.95)	1.26(0.32,4.97)	0.739
PTB(<37 weeks)	1.81(1.05,3.13)	2.40(1.28,4.49)	0.006	1.09(0.70,1.71)	1.17(0.74,1.87)	0.504
VLBW(<1500 g)	2.12(0.41,11.02)	2.65(0.41,17.29)	0.310	1.25(0.31,5.02)	1.32(0.30,5.90)	0.713
LBW(< 2500g)	2.68(1.39,5.18)	2.62(1.27,5.37)	0.009	2.27(1.26,4.07)	1.98(1.06,3.70)	0.032
HBW(>4500 g)	0.88(0.11,7.33)	4.16(0.25,68.21)	0.318	1.67(0.59,4.73)	1.72(0.58,5.12)	0.330
SGA(<10th percentile)	0.60(0.27,1.33)	0.59(0.26,1.36)	0.212	1.70(0.98,2.96)	1.63(0.91,2.90)	0.099
VSGA(<3th percentile)	0.99(0.29,3.43)	0.91(0.25,3.36)	0.888	2.52(0.88,7.24)	2.35(0.76,7.24)	0.138
LGA(>90th percentile)	0.80(0.49,1.30)	0.90(0.54,1.52)	0.702	0.82(0.61,1.11)	0.88(0.64,1.20)	0.417
VLGA(>97th percentile)	1.24(0.61,2.52)	1.58(0.73,3.43)	0.247	0.77(0.49,1.23)	0.81(0.50,1.29)	0.372
Congenital malformations (%)	1.77(0.47,6.60)	1.07(0.26,4.32)	0.929	1.96(0.72,5.29)	1.51(0.54,4.23)	0.432
Neonatal complications(%)	1.06(0.51,2.20)	0.82(0.38,1.76)	0.603	1.50(0.88,2.55)	1.46(0.84,2.53)	0.185
Maternal complications (%)						
GDM	0.87(0.50,1.49)	0.75(0.42,1.33)	0.323	1.34(0.94,1.93)	1.30(0.88,1.91)	0.186
PPROM	2.23(0.78,6.41)	1.86(0.58,6.01)	0.297	1.51(0.65.3.48)	1.35(0.56,3.27)	0.507
Placenta previa	0.94(0.36,2.47)	1.67(0.57,4.90)	0.348	2.27(1.15.4.50)	2.65(1.26,5.57)	0.013
PIH	0.67(0.23,1.93)	0.55(0.17,1.77)	0.317	0.83(0.39,1.78)	0.74(0.32,1.68)	0.469
Pre-eclampsia	1.93(2.67,3.20)	0.81(0.21,3.05)	0.753	1.25(0.50,3.12)	1.17(0.44,3.14)	0.749
Post-partum haemorrahage	1.41(0.46,4.31)	1.13(0.35,3.66)	0.839	0.73(0.27,1.99)	0.61(0.22,1.73)	0.355

*COR, crude odds ratio; AOR, adjusted odds ratio; CI, confidence interval; Analyses were adjusted for maternal age, BMI, parity, FET rank, EM, etiology of infertility, method of endometrial preparation, number of total oocyte, number of MII oocyte, year of treatment, day of transfer, newborn gender.*

The potential risk factors for LBW were shown in Table 5 with unadjusted and adjusted odds ratios (AORs) and 95% confidence intervals (CIs). In the logistic regression model, embryo quality was found to be an independent predictor of LBW (poor quality vs good quality, AOR: 2.41, 95% CI: 1.50–3.86, *p* < 0.001). Moreover, women between 38 and 40 had a significantly higher incidence of LBW than women <35 years (AOR: 2.21, 95% CI: 1.13–4.32, *p* = 0.010) (AOR: 2.21, 95% CI: 1.13–4.32, *p* = 0.010). The thin endometrial thickness on ET day (<8 mm) had a significantly higher risk of LBW compared with the moderate endometrial thickness (8–14 mm) (AOR: 2.70, 95% CI: 1.55–4.71, *p* = 0.001).

**TABLE 5:** COR and AORs of the confounding factors for low birth weight.

**Variable**	**COR (95% CI)**	***P***	**AOR (95% CI)**	***P***
Embryo quality				
Good	Reference	<*0.001*	Reference	<*0.001*
Poor	2.34(1.02,3.61)		2.41(1.50,3.86)	
Maternal age(years)				
<35	Reference	0.016	Reference	0.066
35–37	1.32(0.74,2.34)		1.23(0.68,2.23)	
38–40	2.47(1.33,4.58)		2.21(1.13,4.32)	
Maternal BMI (kg/m^2)				
<24	Reference	0.478		
≥24	1.21(0.72,2.02)			
parity				
0	Reference	0.495		
≥1	1.28(0.63,2.59)			
FET rank				
1	Reference	0.774		
≥2	1.06(0.70,1.63)			
Main infertility cause				
Tubal factor infertility	Reference	0.319		
PCOS	0.81(0.34,1.90)			
Poor ovarian reserve	1.88(0.83,4.25)			
Male infertility	1.05(0.59,1.85)			
Other	0.64(0.31,1.30)			
Endometrial preparation				
Natural cycles	Reference	0.766		
Hormone therapy cycles	1.22(0.70,2.13)			
Stimulated cycles	1.89(0.68,2.21)			
Endometrial thickness on FET day				
8.0–14.0	Reference	0.001	Reference	0.001
<8.0	2.70(1.57,4.63)		2.70(1.55,4.71)	
>14.0	0.57(0.21,1.59)		0.57(0.20,1.60)	
Total oocytes per cycle				
6–15	Reference	0.475		
1–5	1.11(0.71,1.73)			
>15	0.70(0.34,1.45)			
Procedure				
IVF	Reference	0.617		
ICSI	1.00(0.62,1.62)			
IVF+ICSI	0.66(0.28,1.54)			
Day of embryo transfer				
Day 3	Reference	0.815		
Day 5	0.82(0.44,1.51)			
Day 6	0.96(0.61,1.53)			
Year of treatment				
2006–2012	0.95(0.59,1.52)	0.864		
2013–2014	0.84(0.43.1.62)			
2015–2018	Reference			

*AORs were adjusted for all those covariates with adjusted OR in the table using a binary logistic regression model through back ward conditional method.*

## Discussion

This is the first study of FET cycles, exploring the impact of embryo quality on neonatal and maternal outcomes of singletons, and our results demonstrated that the transfer of single PQE was associated with a higher risk of LBW compared with the transfer of single GQE. In cleavage-stage embryo transfer cycles, a poorer neonatal outcome with higher rates of PTB and LBW was detected after the transfer of PQE compared with the transfer of GQE. In the blastocyst transfer cycles, a significantly increased risk of LBW was also noted for PQE transfers, while the difference in PTB was less evident. Therefore, our results, for the first time, reported that the poor embryo quality should be considered as a novel influence factor related to the adverse neonatal outcome.

In line with previous studies (Oron et al., 2014a, b; Zhu et al., 2014; Bouillon et al., 2017), our study further confirmed that embryo quality was associated with lower clinical pregnancy and live birth rates in FET cycles. However, once the clinical pregnancy was achieved, both in the GQE and PQE groups, the live birth rate would reach around 80%, with no significantly increased risks of miscarriage rate and ectopic pregnancies.

To the best of our knowledge, there is a paucity of information on the association between embryo quality and perinatal outcomes in FET cycles. In fresh cycles, however, some studies have demonstrated no significant association between embryo quality and perinatal outcomes. In a study from Canada, singletons were compared retrospectively between 386 single GQE transfers and 54 single PQE transfers and no correlation was detected in any outcome, although the sample size was limited (Oron et al., 2014b). Another observational study from France suggested that poor embryo quality was not associated with adverse obstetrics and perinatal outcomes after single blastocyst transfer, with the singletons further dividing into four groups of good (*n* = 261), fair (*n* = 209), poor (*n* = 109), and early blastocyst quality (*n* = 72) (Bouillon et al., 2017). The same conclusion was obtained by Zhu et al. (2014) when 2586 singletons born after double cleavage embryos transfer were compared between GQE (*n* = 2487) and PQE (*n* = 99) (Zhu et al., 2014). Interestingly, a cohort study including a total of 224 newborns resulting from single fresh and frozen blastocyst transfers demonstrated that a higher grade of ICM was associated with greater birth weight compared with those with less advanced ICM in fresh cycles, though the observation did not present in frozen cycles (Luke et al., 2017). The discrepancies between those studies may be restricted to diverse definitions of embryo quality and the design of fresh embryo transfer itself.

During the embryo culture period, disparities in embryo quality are routinely seen in individual embryo cohorts, and many factors were related to poor embryo quality, including patient-specific parameters such as parental advanced age, maternal obesity, and poor ovarian reserve infertility (van Rooij et al., 2003; Check et al., 2007; Shah et al., 2011; Dobson et al., 2018), which might also be predisposed to adverse perinatal outcomes (Pelkonen et al., 2010; Wennberg et al., 2016). A large population-based study included all 16 IVF clinics in Sweden, involving cleavage-stage embryo transfer and blastocyst transfer, found significantly higher rates of PTB and LBW births in non-elective SET singletons compared with elective SET, probably due to some of the embryos in non-elective SET were PQEs (Sazonova et al., 2011), which was in accordance with our results to some extent, though the detailed information about embryo quality was not specified in the non-elective SET group.

Furthermore, our study would benefit from the improved synchrony between embryos and the uterine environment following FET as the supraphysiologically high hormone levels in fresh cycles were removed (Pelkonen et al., 2010; Pinborg et al., 2013; Sha et al., 2018). Alternatively, the effect of frozen-thawed procedures has been postulated through the aberration of DNA methylation and the expression of multiple growth-related imprinted genes (Young et al., 1998; Khosla et al., 2001), which may in turn affect fetal development. However, the exact mechanisms behind the observed neonatal differences in our study are still to be elucidated. It remains unclear whether such outcomes might be due to the vitrified-warmed process itself or to impaired potential in PQEs. In the present study, we also found an increased risk of placenta previa after the transfer of poor quality blastocyst (4.5 vs 2.3%). We supposed that the poor embryo quality might also be associated with abnormal placentation, and then in turn partially explain the higher incidence of LBW. However, the findings should be interpreted with caution due to the low prevalence of maternal complications in the study population.

Moreover, It has been suggested that increased risks of LBW and PTB were correlated with maternal characteristics, such as maternal factor infertility (Wennberg et al., 2016) and maternal age (Sazonova et al., 2011). Previous studies have indicated that poor ovarian response was related to decreased embryo morphology grades and increased perinatal risks (Pelkonen et al., 2010; Wennberg et al., 2016; Morin et al., 2018), and in the current study, we also noticed a higher proportion of poor ovarian reserve infertility in women with PQE transferred, though the difference did not reach statistical significance. Advanced maternal age in pregnancy was also associated with increased perinatal risks including hypertensive disorders, placenta previa, PTB, LBW, and SGA. A large registry study based on data from national ART registries, covering the years 1982–2007, observed that the risk of adverse neonatal outcome was significantly higher in ART singletons for most maternal ages, compared with singletons conceived spontaneously.

It is also reported that the formation of ice crystals in the procedure of freezing and thawing can reduce embryo quality (Mandelbaum et al., 1998), however, the advent method of embryo vitrification used in the current study have accomplished a survival rate around 99% with the absence of ice crystals (Cho et al., 2002; Kuwayama et al., 2005), and the quality of embryos post-thawed was used as the variable in our study, which was the same as the embryo quality before vitrification.

Additionally, certain studies focused on embryonic growth have shown that embryo quality was closely associated with embryo development speed and euploid rates (Capalbo et al., 2014; Du et al., 2018; Irani et al., 2019). The current study showed a higher proportion of growth-delayed day 6 embryo in the PQE. Likewise, Du et al. (2018) have demonstrated that the allocation of inferior morphological grades increased by the order of day 5, day 6, and day 7, and they found no significant difference in the rate of LBW between day 5, day 6, and day 7, however, the analysis was conducted without controlling potential confounders. Poor embryo morphology was also concerned with damaged embryonic euploid viability, though the negative effect of aneuploidy was reported mainly realized in failed implantation and clinical loss, while less resulted in affected babies (Capalbo et al., 2014; Irani et al., 2019). We also found that the proportion of neonatal complication or congenital malformations was slightly higher in singletons derived from PQE than singletons derived from GQE, but significant differences were not observed.

It is important to note that the present study combined SET with FET, removed the effect of vanishing twin phenomenon, which was significantly associated with higher risks of PTB and LBW (Kamath et al., 2018). Another major strength is that the study was performed in a single center where embryos were graded by the same group of trained embryologists, avoiding the argument of different embryo quality criteria from multiple centers. The strict individual eligibility criteria utilized in our study also limit the bias of advancing age and obese patients, who were also at higher risk of adverse neonatal outcomes.

Our study was limited by the retrospective nature, which including the absence of some information on potential confounders such as maternal smoking status, but the proportion of smoking women was quite few in China^1^. The other limitation was the follow-up telephone interview, which was less accurate than the abstraction of medical records.

In conclusion, the ultimate test of embryo quality is the promise of a healthy pregnancy that progresses to live birth. Our findings could raise awareness to clinicians consulting patients on the chance of delivering a healthy normal-weight baby when there are only PQEs available for transfer. The effect of embryo quality warrants further investigations by larger registry studies to obtain a more accurate understanding of the relationship between embryo quality and obstetric and perinatal outcomes.

## Data Availability Statement

All datasets generated for this study are included in the article/Supplementary Material.

## Ethics Statement

The studies involving human participants were reviewed and approved by Institutional Review Board of the Shanghai Ninth People’s Hospital. The patients/participants provided their written informed consent to participate in this study.

## Author Contributions

YW supervised the entire study, including the procedures, conception, design and completion. JH, YT, JZ, XY, JW, and YK were responsible for the collection of the data. JH contributed the data analysis and drafted the manuscript. YW participated in the interpretation of the study data and revisions to the manuscript.

## Conflict of Interest

The authors declare that the research was conducted in the absence of any commercial or financial relationships that could be construed as a potential conflict of interest.

## Footnotes

1 http://news.xinhuanet.com/fortune/2013 –05/31/c_124794069.htm
