# Supplementary material for: Poor Embryo Quality Is Associated With A Higher Risk of Low Birthweight in Vitrified-Warmed Single Embryo Transfer Cycles
Source: Front Physiol. 2020 May 15;11:415. doi: 10.3389/fphys.2020.00415 (PMC7243353; doi:10.3389/fphys.2020.00415)
Supplement: Supplementary file 1 [file Table_1.pdf]

Table S1-Types of congenital malformations among 2403 live born singletons.

Malformation type	ICD-10 code and diagnosis (n)	‰(n/N)
Circulatory system	Q21.0: ventricular septal defect (2); Q21.1: atrial septal defect (3); Q21.3: tetralogy of Fallot (1); Q25.6: stenosis of pulmonary artery (1)	7(2.9)
Digestive system	Q31.8: congenital larygomalacia (1); Q35.9: cleft palate (1); Q41.0: congenital duodenal atresia (1); Q43.3: congenital intestinal malformation (1)	4(1.7)
Urinary system	Q62.0: congenital hydronephrosis (1)	1(0.4)
Musculoskeletal system	Q65.8: congenital hip dysplasia (2)	2(0.8)
Nervous system	Q03.9: congenital hydrocephalus (1); Q37.8: demyelinating Encephalopathy (1)	2(0.8)
Other congenital malformation	Q11.1: congenital anophthalmia(1); Q28.8: congenital aneurysm (1); Q82.5: vascular nevus (2)	4(1.7)
Birth defects total		20(8.3)

Note: ICD-10 = International Classification of Diseases, 10th edition.
